# Adenosine triphosphate (ATP) sampling algorithm for monitoring the cleanliness of surgical instruments

**DOI:** 10.1371/journal.pone.0284967

**Published:** 2023-08-15

**Authors:** Daniela Oliveira Pontes, Dayane de Melo Costa, Priscilla Perez da Silva Pereira, Greg S. Whiteley, Trevor Glasbey, Anaclara Ferreira Veiga Tipple

**Affiliations:** 1 Faculty of Nursing, Federal University of Goiás, Catalão, Brazil; 2 Nursing Department, Federal University of Rondônia, Porto Velho, Brazil; 3 Faculty of Medicine, Health and Human Sciences, Macquarie University, Sydney, Australia; 4 Faculty of Medicine and Health, University of Sydney, Sydney, Australia; 5 School of Medicine, Western Sydney University, Penrith, Australia; 6 Whiteley Corporation, Kewdale, Australia; Jeonbuk National University, REPUBLIC OF KOREA

## Abstract

**Background:**

Timely detection of cleaning failure is critical for quality assurance within Sterilising Service Units (SSUs). Rapid Adenosine Triphosphate (ATP) testing provides a real time and quantitative indication of cellular contaminants, when used to measure surface or device cleanliness. The aim of this study was to investigate the use of an ATP algorithm and to whether it could be used as a routine quality assurance step, to monitor surgical instruments cleanliness in SSUs prior to sterilisation.

**Methods:**

Cleanliness monitoring using rapid ATP testing was undertaken in the SSUs of four hospitals located in the western (Amazonia) region of Brazil. ATP testing was conducted (Clean Trace, 3M) on 163 surgical instruments, following manual cleaning. A sampling algorithm using a duplicate swab approach was applied to indicate surgical instruments as (i) very clean, (ii) clean, (iii) equivocal or (iv) fail, based around a ‘clean’ cut-off of 250 Relative Light Units (RLU) and a ‘very clean’ <100 RLU.

**Results:**

The four cleanliness categories were significantly differentiated (P≤0.001). The worst performing locations (hospitals A & C) had failure rates of 39.2% and 32.4%, respectively, and were distinctly different from hospitals B & D (P≤0.001). The best performing hospitals (B & D) had failure rates of 7.7% and 2.8%, respectively.

**Conclusion:**

The ATP testing algorithm provides a simple to use method within SSUs. The measurements are in real time, quantitative and useful for risk-based quality assurance monitoring, and the tool can be used for staff training. The four-tiered approach to the grading of surgical instrument cleanliness provides a nuanced approach for continuous quality improvement within SSU than does a simple pass/fail methodology.

## Introduction

The effective cleaning of Surgical Instruments (SIs) and other reusable medical devices is the essential first step in the reprocessing of these devices prior to re-use. Cleaning failure represents a hazardous compromise to subsequent sterilisation processes. The introduction of a timely, reliable and simple to use quality assurance step to detect the presence of contamination, that has evaded the cleaning processes, will be advantageous for Sterilising Service Units (SSUs) worldwide.

The detection of Adenosine Triphosphate (ATP), using rapid ATP testing equipment and consumables, is a quantitative and real time approach to cleaning monitoring that has been initially trialled for use as a cleanliness indicator within SSUs [1]. The failure of cleaning processes for reusable medical devices presents an infection risk for patients [2]. Various issues may compromise the outcome of sterilisation process, not the least of which may be biofilms containing viable and resistant pathogenic microorganisms, or wear on an SI surface, or even drying time awaiting reprocessing, and so cleanliness monitoring is a critically important issue for sterilising departments globally [3–5].

There is literature in support of the use of ATP testing for cleanliness monitoring in applications, including healthcare, food manufacturing and surface hygiene [6, 7]. Cleanliness testing is an advantage for those who wish to lower the risk of cleaning failure, which in turn can lead to SI sterilisation failure. Cleanliness testing is a logical quality assurance step for use in SSUs, and rapid ATP testing offers clear opportunities as a quick and easy to use method for that purpose.

There are published studies attempting validation of rapid ATP devices and consumables, but with mixed results [8, 9], thus there are controversies regarding the use of rapid ATP testing for the purpose of cleanliness testing [10, 11]. One of the critical in-use issues is the scaling in Relative Light Units (RLUs), as this is a truly relative scale that prevents any interoperability or quasi-relationship between the results produced by the different ATP branded devices, even when tested on same soils [8, 9]. Selection of the brand of ATP device requires a clear understanding of its dynamic range and capacity to produce meaningful results, especially at the lower limit of quantitation.

There are also problems with variability and imprecision, as the risk of a false result is nearly 20% where only a single swab sample is taken [12]. This inherent variability is so high that with any result that relies on just a single swab sample, there is a 20% chance that the reading could be wrong by a factor of two. The use of a poor sampling approach, and a reliance on single swab for sampling of each surface or device can undermine statistical validity for any results [13].

Whiteley et al. have proposed a sampling algorithm for ATP testing, which was applied to assess the cleanliness of ultrasound equipment surfaces, and indicated a high level of safety of the results, mitigated the error inherent to the devices and sampling, as well as pointed to the possibility of its use for other reusable medical devices [14, 15]. This method has not been applied for use in cleanliness testing of SI or other reusable medical devices prior to sterilisation. An updated version of the ATP algorithm has also been published, but without follow up studies [16].

In this study, the ATP algorithm was applied for cleanliness monitoring of SI following manual cleaning and prior to sterilisation. The purpose of the study was to investigate the use of this ATP algorithm and to whether it could be used as a routine quality assurance step, to monitor SI cleanliness in SSUs prior to sterilisation.

## Methods

Four hospitals, located in the north region of Brazil, State of Rondônia, were included in this study, and were identified only as hospitals A, B, C & D. The ATP testing was performed by the researchers, in the packing area of the SSUs. A total of 163 SI was selected, including various forceps & hinged instruments (e.g. adson, bakey clamp, collin, crille, dissection, foester, guyon, kelly, kocher, mixer, mosquito, pean), urology biopsy forceps, needle holder and scissors. The SI were selected at the packing area according to the SSU demand for each hospital, and included in the study those SI that met the following criteria: surface area of >20cm^2^ and compatibility for swab collection. The sampling area was guided by a 10 cm x 1cm (length x wide) paper template. Gloves were used to handle the SI, and the ATP swab were fractioned in rotation movement (back and forth) from the proximal (rod) to the distal (tip) of the SI.

In this study, two duplicate samples were taken from the surface of each of the SI following a manual cleaning process, but prior to sterilisation. The duplicate ATP measurements from each SI were then recorded as a matched pair (samples1 & 2). The use of duplicate samples does increase the number of swabs being used, but it plays a critical role in mitigating the impact of inherent variability with the ATP measurement devices, reducing the error from 20% per sample/item to less than 1% per item [16]. The use of two swabs is recommended in the algorithm method, over the use of a single swab over a larger surface area, as the risk of variance in any one swab is discreet, and the application of larger surface area will not mitigate the possibility of variance, as well as the use of a second and discreet swab.

In the first iteration of the method, where there was doubt about either of the initial duplicate readings, additional ATP measurements were taken [14]. The readings were then classified as per the more recent and simplified ATP algorithm, and only samples 1 & 2 were used to determine the cleanliness category that applied to each item [16].

For this study, Swab Clean Trace Surface ATP device (HX0012-00159, 3M Corporation, USA) was used. As recommended in the ATP algorithm method, a clean threshold of 250 RLU was set as the pass/fail level, and a second tier very clean level was set at 100 RLU [14]. Where both samples were less than 100 RLU, the discreet classification of “very clean” was applied. Where both of the samples were below 250 RLU (in some instances one sample was less than 100 RLU, but the other sample was less than 250 RLU), then the “clean” classification applied. Where one sample was below 250 RLU, but the other sample greater than 250 RLU, then the classification of “equivocal” was applied. Where both ATP samples were greater than 250 RLU, then the classification of “dirty/fail” was applied. The frequency of this measurement is a critical risk issue and represents a failure of cleaning in the removal of cellular soils, as a surrogate for overall cleanliness.

The outline of the cleanliness tiers is set out in Table 1. Risk classes are attributed colour codes in alignment with best practice for ease of recognition of the risk rating [16].

**Table 1:** Cleanliness classifications based on Relative Light Unit (RLU) measurements from paired samples.

Cleanliness classification	RLU levels on measurement	Action
**Very clean**	Both < 100 RLU	-
**Clean**	Both < 250 RLU	-
**Equivocal**	One < 250 RLU + One > 250 RLU	Re-cleaning according to the 3^rd^ or 4^th^ sampling result*.
**Dirty/Fail**	Both > 250 RLU	Re-cleaning*.

*According to Witeley et al. [16]

The use of a tiered scale for cleanliness testing with rapid ATP tests has been outlined previously with based field testing of gastroendoscopes [17, 18]. However, a single point, pass or fail approach based around a single fixed measurement, using only a single ATP test per sample, is beyond the precision level of these field based devices [12]. A three-tiered approach–with a pass, alert and action level–is adopted in BS EN ISO 15883‑5:2021, although the primary purpose of that standard is in the type testing and validation of washer disinfectors in a laboratory environment, with known quantities of defined test soils, where ATP testing is available as a second analyte option [19]. Field based SIs cleanliness testing is not an anticipated use of that standard.

For this field-based study of SIs cleanliness following a manual cleaning process inside of SSU, where contemporaneous validation is limited to positive control confirmation, the use other analytes was not possible, particularly in a remote region of the Western Amazon (State of Rondônia).

Standard statistical processes were used for the tabulation, analysis, calculations, table and figures were completed using Microsoft Excel 2016 (Microsoft Systems, WA USA), and some statistical analysis was completed using Sigma Plot for Windows, v14, 2017 (Systat Systems, CA, USA). P-value < 0,05 were considered statistically significant.

## Results

**Fig 1:**
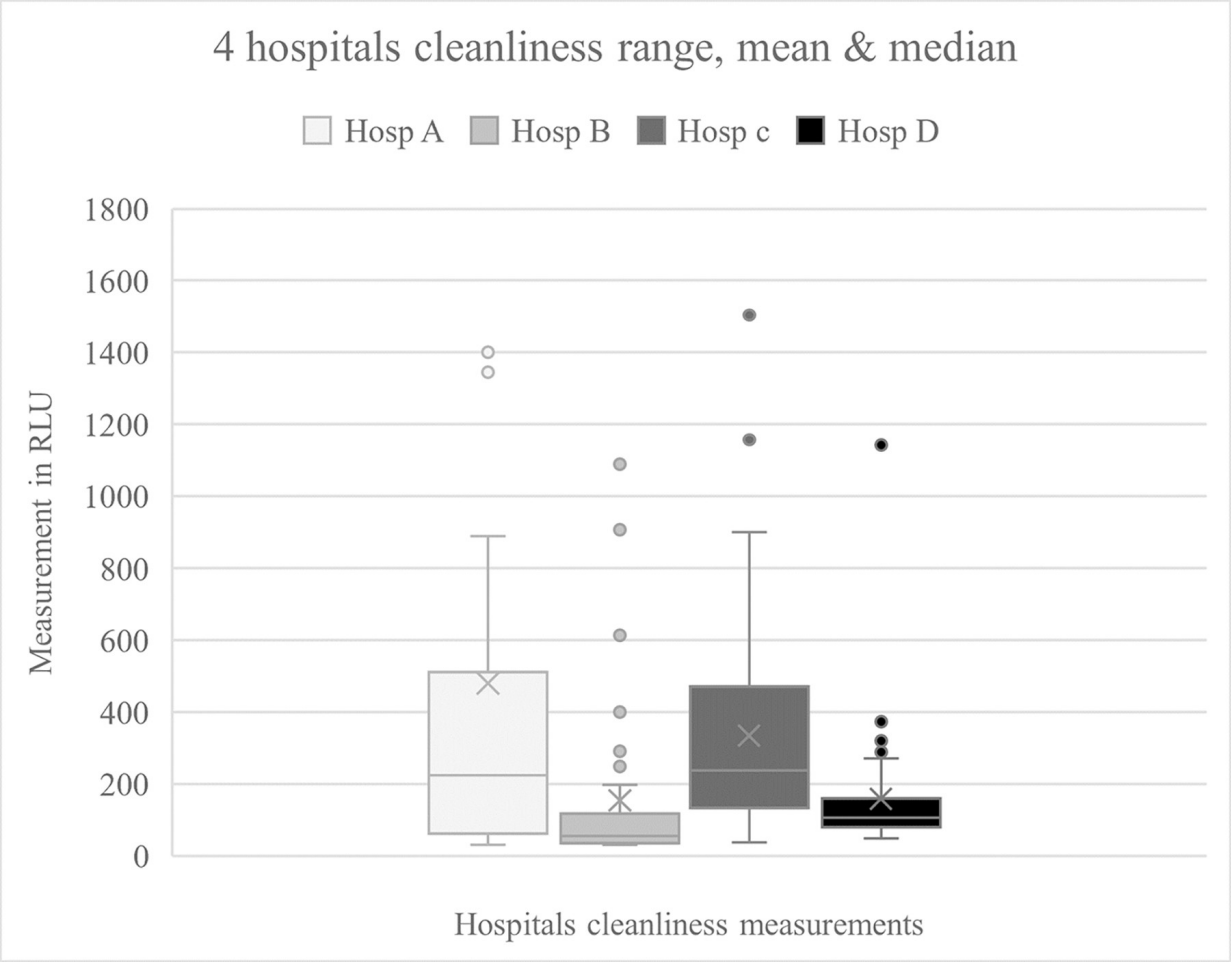
Data cleanliness measurements: Variance and spread. RLU–Relative light unit.

The data sets of results from cleanliness testing at each of the four hospitals is shown in Fig 1, set up as a box and whisker plot. The data for hospital A includes two very high outliers, which are not included on the graph as shown. The data from each of the hospitals lacks normality, and so some standard tests were not applicable for determining the significance of association within the data sets.

From Fig 1, it is observed that for hospitals A & C, the data range and inter-quartile ranges are quite similar. The similarity of the data set from hospitals B & D is also striking in appearance with their very condensed inter-quartile range, although there are also outliers for both of these hospitals. The data sets of hospitals A & C were significantly distinct from the data sets from the other two hospitals (B & D), using a multiple pairwise comparison (P<0.001, Bonferroni t-test).

**Fig 2:**
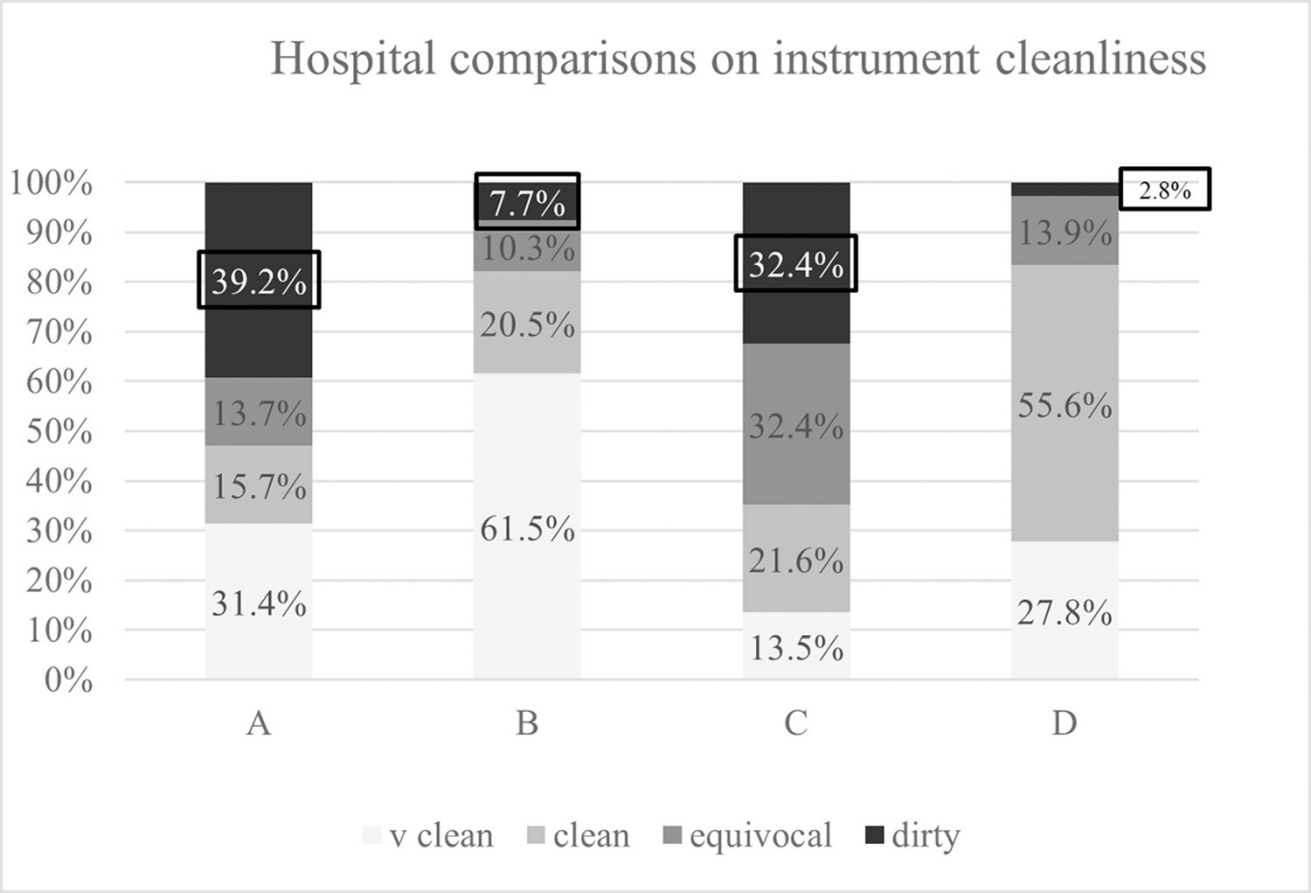
Comparing hospital performance on surgical instruments cleanliness. v clean–very clean. RLU—Relative Light Units.

The mean from each pair of readings from each individual SI were classified as per table I in S1 File. For each hospital, the individual number of SI attributed to each of the four-tiered cleanliness classification is shown. For ease of presentation, this data was then converted to percentages and is graphically displayed with risk colour coding in Fig 2.

The two highest failure levels are recorded for hospitals A & C (39.2% and 32.4% respectively). The data viewed from Fig 2 strengthens the observation that hospitals A & C display similarity in data proportions and that hospitals B & D have similar proportions. Whereas, hospitals A & C, when compared with Hospitals B & D, are quite dissimilar. Both Hospitals B & D had total clean frequencies (combined very clean and clean categories) of 82.0% and 83.3%, respectively. Hospitals A & C had combined fail/dirty & equivocal rates of 52.3% and 64.9%, respectively.

Multiple pairwise analysis on the strength of association between the hospitals showed no significant difference between hospitals B & D (P = 0.473 Bonferroni t-Test), and similarly when cross checked using a rank sum of squares test for hospitals A & C (P = 0.658 Mann-Whitney). When mean of pairs from the SI from hospitals A & C is aggregated and then compared with the mean of pairs aggregated data from hospitals B & D, the data is significantly differentiated (P≤0.001, One Way ANOVA, Dunn’s method).

The overall outcome for each of the cleanliness categories is for ‘very clean’, 34% (55/163); for ‘clean’ 27% (44/163); for the ‘equivocal’ group 17% (28/163); and for the ‘dirty/fail’ category 22% (36/163) (Fig 2).

As for those SI classified as "dirty" (22%), the highest RLU values were for the urology biopsy forceps (SSU hospital A), bakey clamp forceps (SSU hospital B), dissection forceps (SSU hospital C), and dissection forceps and scissors (SSU hospital D). The ATP values found in urology biopsy forceps points to the challenge and the need of careful cleaning of complex-design SI, as it makes access to its entire area difficult.

The percentages for each classification for each of the four hospitals is shown in Fig 2, which displays the percentages to normalise the data. The poor cleanliness of SI for hospitals A & C, where both have > 30% level of failure is also shown in red in Fig 2. The cleanliness performance of hospitals B & D is superior for the categories of “very clean” and “clean”, but only if they are combined into a single grouping.

**Fig 3:**
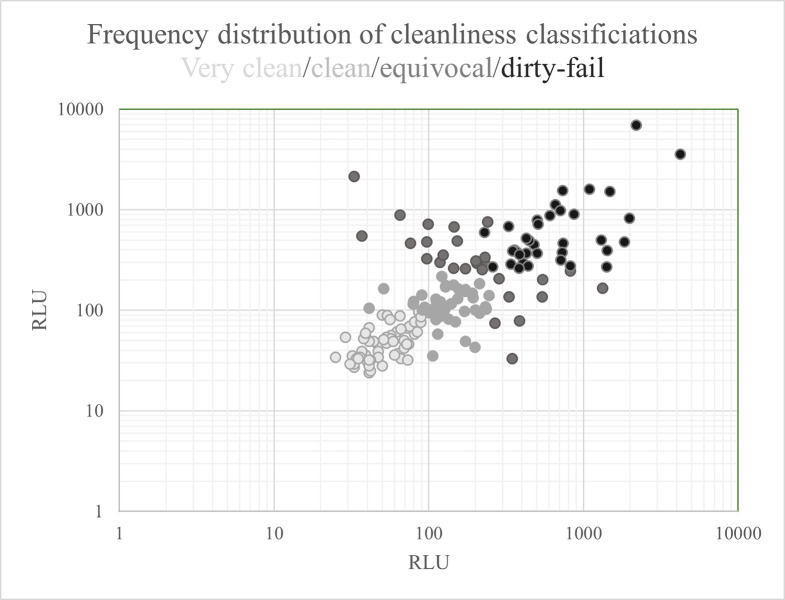
Samples 1 and 2 combined as an X & Y plot. RLU—Relative Light Units.

The data from the four classification categories is plotted by the pairs of samples (Fig 3), showing the separation between the categories.

The pairs of samples were subjected to testing to determine if the cleanliness classifications were sufficiently independent. Using a one-way analysis of variance on ranks demonstrated that all four cleanliness classifications data sets were all statistically different (P≤0.001, Kruskal-Wallis). Additional testing using a pairwise multiple comparison method indicated all classifications were differentiated, excepting the two classifications of ‘equivocal’ and ‘dirty/fail’, which lacked difference (P<0.284, Dunn’s method). This is unsurprising as at least one reading from each pair is in excess of the 250 RLU cut-off.

The data for the cleanliness categories was then amalgamated into just two categories of clean (amalgamating very clean & clean) or dirty/fail (amalgamating equivocal & dirty/fail) for each of the four hospitals. This treats the ATP testing approach as binomial on a pass or fail basis for quality assurance purposes. The difference between the pass (clean) grouping and fail (E & D/F) grouping is significant for each hospital in the study (P≤0.001, Mann Whitney).

## Discussion

The application of the algorithm to critical SI after cleaning was simple, uniform, added ease in the evaluation and possibility of comparing the results between different SSU. The use of duplicate swabs was easy to do in the SSUs, and only added a small amount of additional time to the sampling and the cost of one additional swab. This identified 28 SI (17%) that might otherwise have been mis-classified.

The identification of SI with residual soiling (which may include live cells such as microorganisms), in real time, allowed immediate, targeted and systematic intervention of the cleaning process in the units evaluated, aiming continuous improvement of the reprocessing. The immediate feedback provided by ATP testing also allows an important educational aspect for staff involved in the cleaning and quality assurance processes within SSU. The use of a quantitative method such as ATP testing is superior in safety and quality compared to notoriously unreliable visual inspection methods [20].

An experimental laboratory study that evaluated two cleaning methods, manual and manual followed by automated, showed that the two cleaning methods reduced the amount of ATP, however, only in SI subjected to manual method had biofilm [21]. This reinforces the cleaning monitoring recommendations as cleaning is recognized as the most important reprocessing step, thus essential to the success of further steps [22].

The ATP algorithm normally includes a Cleaning Intervention Step (CIS) to ensure that the surfaces can in fact be effectively cleaned, however, for this quality assurance study with SI and other reusable medical devices, it was assumed that cleaning of all SI could be achieved, if conducted effectively. The CIS was not done which saved on swab numbers and treated monitoring purely as a quality assurance step.

We know that cleaning failure can lead to sterilisation failure [6, 23, 24]. The data provided in this study allowed for a nuanced approach to risk managing cleaning failure in SSU. If the cleanliness is assessed into four categories of cleanliness as per table I in S1 File, then a traditional colour coded risk approach can be adopted. This multi-tiered approach allows staff to engage in program of quality assurance improvements, whilst aiming to achieve the attainable goal of very clean instruments prior to sterilisation. The 100 RLU threshold for very clean sits just above the identified Lower Limit of Quantitation (LLQ) for this branded ATP device [9]. Regular monitoring of SI cleanliness in SSU, using a method that colour codes from reliable and quantitative ATP measurements, allows staff to visualise cleaning performance and the see improvement opportunities.

The ‘alert’ and ‘action’ levels of ATP set for the ISO 15883–5:2021 standard are referenced and based on information available at the time of writing the standard.

In our field measurements, we have used a method that has been validated to mitigate for inherent variance with the ATP testing equipment, and which improves the sampling rationale to provide a statistically consistent outcome (based on laboratory validations) [14]. The ATP algorithm method also has a more nuanced approach to the three tiers, which mitigates the potential inherent variance through use of a duplicate initial sample [16]. Use of a three-tiered approach without mitigating for the inherent variance is likely to be statistically flawed [14]. Clearly, SIs which score above the clean threshold should be immediately withdrawn from use and subjected to reprocessing and re-testing. We suggest that any equipment that is classed as ‘equivocal’ should also be subjected to reprocessing.

The clean threshold of 250 RLU was originally proposed for use with surface hygiene [25]. The proposal to move to 200 RLU as a threshold is based around studies on cleaning of gastroendoscopes, using a different method involving a flush sample from the entire length of an endoscope, following a manual cleaning process and uses a different type of ATP sampling from a liquid sample [17]. The field study in support of this lower 200 RLU threshold used the same method, using different swabs intended solely for use with liquid samples [18]. This current research used swabs intended solely for surface sampling. The real difference between these two readings of 200 and 250 RLU is nether right nor wrong, but for this study using direct to surface swabs, the 250 RLU cleanliness cut-off was preferred.

The slightly shortened ATP algorithm can also be used as a binomial pass/fail system with a hard threshold. Whilst this approach was statistically defensible for this study, there remains doubt over the precision of ATP testing equipment, so that the defined threshold is somewhat arbitrary. The role of the equivocal data set is to identify a risk that might be overlooked with a single sample, but the problem really lies with the clean data set where there may be two readings close to, but just under the threshold cut-off, which for this study was 250 RLU. One cannot realistically challenge the association between two identical SI, where one item scores two readings at say 249 RLU, whereas the other identical SI records two readings at say 251 RLU, is there really a distinguishable difference between the results? [12]

The use of four tiers also allows for a more graded approach to continuous quality improvements, particularly when used over a period of time. Key performance indicators can be set around each of the four categories so that improvements in cleaning outcome are shown in diminishing numbers of “fail” or “re-clean”, and improvements are made to the frequency of “clean” or “very clean”.

This study could have been improved with additional microbiology to interrogate the actual microbial cleanliness in a quantitative manner, and this may be done with additional resources in a follow up study. Conversion to a quantified scale, such as Femtomoles, may be possible with further qualification from the equipment manufacturer. However, in remote regions conversion is not possible without an accurate and quantitative positive control tool, not available for this study. Using an assumed conversion to Femtomoles would not be reliable without additional manufacturer on-site validation support. The study was conducted in four regional hospitals in western Brazil, and there is no certainty that the same outcomes for actual SI cleanliness would be achieved from larger capital city, based full tertiary hospitals within a fully urban environment. It would also have been interesting to include matched SI types between the four hospitals to really achieve comparative cleanliness performances.

## Conclusion

For this study, the ATP algorithm was a very useful tool that enabled a simple to apply and nuanced approach to measuring SI cleanliness. This study supports a view that with an improved sampling approach as used here, whilst there may be more ATP swabs used, the data outcomes have greater validity and provide a more reliable quality assurance outcome for SSU managers. The improvement in data reliability from the use of the algorithm method is important for an evidenced-based approach to reusable medical device cleanliness within health care settings globally.

## Supporting information

S1 File (PDF)
