# Supplementary material for: Adenosine triphosphate (ATP) sampling algorithm for monitoring the cleanliness of surgical instruments
Source: PLoS One. 2023 Aug 15;18(8):e0284967. doi: 10.1371/journal.pone.0284967 (PMC10426997; doi:10.1371/journal.pone.0284967)
Supplement: S1 File — (PDF) [file pone.0284967.s001.pdf]

Title: Adaptation of an adenosine triphosphate (ATP) sampling algorithm for monitoring the cleanliness of surgical instruments following manual cleaning

Database legend

- identification: number of identification of the surgical instrument
- Dirty: hospital identification: 0 – hospital B; 1 – hospital A; 2 – hospital C; 3 – hospital D
- typinstru: Name of the surgical instrument: 0 – dissecation; 1- crille; 2- scissor; 3- allis; 4 – mosquito; 5- needle holder; 6 – Kocher; 7- assepsia/Pean; 8 – Collin; 9 – Kelly; 10 – Guyon; 11 – Adson; 12 – urology biopsy forceps; 13 – clamp de Bakey; 14 – Mixter; 15 – Foester
- classinstru: classification of the surgical instrument: 0 – exeresis; 1 – hemostatic
- design: classification of the surgical instrument design: 0 – simple; 1 – complex
- atp1t: 1st ATP test made: 0 – yes; 1 -no
- atp1: reading 1st ATP test
- atp2t: 2nd ATP test made: 0 – yes; 1 -no
- atp2: reading 2nd ATP test
- atp3t: 3rd ATP test made: 0 – no; 1 – yes
- atp3: reading 3rd ATP test
- atp4t: 4th ATP test made: 0 – no; 1 – yes
- atp4: reading 4th ATP test
- atpclassfin: classification of surgical instrument according to the ATP test reading: 0 – very clean; 1 – dirty; 2- clean

Results database

identification	hosp	typinstru	classinstru	design	atp1t	atp1	atp2t	atp2	atp3t	atp3	atp4ft	atp4	atpclassfin
1	1	1	1	0	0	47	0	45	0		0		0
2	1	1	1	0	0	128	0	171	0		0		2
3	1	1	1	0	0	44	0	49	0		0		0
4	1	1	1	0	0	113	0	123	0		0		2
5	1	1	1	0	0	41	0	67	0		0		0
6	1	1	1	0	0	1982	0	821	0		0		1
7	1	1	1	0	0	357	0	397	0		0		1
8	1	1	1	0	0	50	0	28	0		0		0

9	1	1	1	0	0	62	0	61	0		0		0
10	1	10	1	0	0	74	0	46	0		0		0
11	1	10	1	0	0	57	0	56	0		0		0
12	1	10	1	0	0	47	0	39	0		0		0
13	1	10	1	0	0	121	0	216	0		0		2
14	1	10	1	0	0	371	0	379	0		0		1
15	1	10	1	0	0	127	0	103	0		0		2
16	1	10	1	0	0	348	0	387	0		0		1
17	1	10	1	0	0	4228	0	3579	0		0		1
18	1	11	1	0	0	65	0	88	0		0		0
19	1	11	1	0	0	866	0	905	0		0		1
20	1	11	1	0	0	229	0	598	1	344	0		1
21	1	11	1	0	0	99	0	715	1	127	0		1
22	1	11	1	0	0	480	0	450	0		0		1
23	1	11	1	0	0	86	0	96	0		0		0
24	1	11	1	0	0	1090	0	1600	0		0		1
25	1	12	1	1	0	386	0	78	1	198	0		2
26	1	12	1	1	0	236	0	103	0		0		2
27	1	12	1	1	0	407	0	293	0		0		1
28	1	12	1	1	0	740	0	462	0		0		1
29	1	12	1	1	0	2193	0	6926	0		0		1
30	1	12	1	1	0	660	0	1118	0		0		1
31	1	12	1	1	0	438	0	275	0		0		1
32	1	12	1	1	0	1417	0	270	1	336	0		1
33	1	13	1	0	0	63	0	37	0		0		0
34	1	13	1	0	0	47	0	34	0		0		0
35	1	13	1	0	0	29	0	54	0		0		0
36	1	13	1	0	0	25	0	34	0		0		0
37	1	13	1	0	0	73	0	66	0		0		0
38	1	13	1	0	0	53	0	47	0		0		0
39	1	13	1	0	0	134	0	82	1	128	0		2
40	1	0	1	0	0	825	0	247	1	130	0		1
41	1	0	1	0	0	502	0	367	0		0		1
42	1	0	1	0	0	172	0	258	1	118	0		2

43	1	0	1	0	0	156	0	162	0		0		2
44	1	2	1	0	0	231	0	289	1	113	0		2
45	1	2	1	0	0	605	0	877	0		0		1
46	1	1	1	0	0	111	0	129	0		0		2
47	1	4	1	0	0	822	0	277	0		0		1
48	1	4	1	0	0	327	0	679	0		0		1
49	1	4	1	0	0	284	0	205	1	238	0		2
50	1	4	1	0	0	1332	0	165	1	200	0		1
51	1	4	1	0	0	503	0	787	0		0		1
52	3	0	1	0	0	153	0	487	1	1057	0		1
53	3	0	1	0	0	191	0	148	0		0		2
54	3	5	1	0	0	113	0	101	0		0		2
55	3	0	1	0	0	112	0	124	0		0		2
56	3	2	1	0	0	84	0	61	0		0		0
57	3	5	1	0	0	114	0	58	1	71	0		0
58	3	0	1	0	0	66	0	65	0		0		0
59	3	0	1	0	0	97	0	326	1	112	0		2
60	3	2	1	0	0	76	0	466	1	112	0		2
61	3	5	1	0	0	81	0	77	0		0		0
62	3	0	1	0	0	90	0	142	1	83	0		2
63	3	0	1	0	0	734	0	1550	0		0		1
64	3	1	1	0	0	56	0	81	0		0		0
65	3	2	1	0	0	544	0	202	1	138	0		1
66	3	0	1	0	0	39	0	59	0		0		0
67	3	0	1	0	0	41	0	105	1	33	0		0
68	3	0	1	0	0	51	0	51	0		0		0
69	3	5	1	0	0	72	0	46	0		0		0
70	3	7	1	0	0	59	0	49	0		0		0
71	3	0	1	0	0	91	0	101	1	88	0		0
72	3	0	1	0	0	212	0	93	1	120	0		2
73	3	0	1	0	0	200	0	101	0		0		2
74	3	5	1	0	0	104	0	102	0		0		2
75	3	2	1	0	0	94	0	107	1	3047	1	79	1
76	3	2	1	0	0	119	0	121	0		0		2

77	3	1	1	0	0	170	0	97	1	64	0		2
78	3	1	1	0	0	129	0	177	0		0		2
79	3	1	1	0	0	101	0	94	1	70	0		0
80	3	1	1	0	0	145	0	178	0		0		2
81	3	1	1	0	0	109	0	91	1	67	0		0
82	3	1	1	0	0	107	0	103	0		0		2
83	3	1	1	0	0	89	0	76	0		0		0
84	3	1	1	0	0	233	0	108	0		0		2
85	3	1	1	0	0	140	0	116	0		0		2
86	3	1	1	0	0	97	0	479	1	102	0		2
87	3	1	1	0	0	89	0	86	0		0		0
88	0	10	1	0	0	41	0	24	0		0		0
89	0	14	1	0	0	37	0	546	1	32	1	31	2
90	0	14	1	0	0	42	0	25	0		0		0
91	0	6	1	0	0	33	0	27	0		0		0
92	0	6	1	0	0	40	0	31	0		0		0
93	0	1	1	0	0	50	0	90	0		0		0
94	0	1	1	0	0	118	0	87	0		0		0
95	0	11	1	0	0	53	0	54	0		0		0
96	0	0	1	0	0	33	0	29	0		0		0
97	0	0	1	0	0	79	0	115	1	82	0		0
98	0	2	1	0	0	69	0	42	0		0		0
99	0	2	1	0	0	41	0	49	0		0		0
100	0	2	1	0	0	57	0	52	0		0		0
101	0	6	1	0	0	32	0	35	0		0		0
102	0	0	1	0	0	66	0	33	0		0		0
103	0	1	1	0	0	173	0	49	1	35	0		0
104	0	6	1	0	0	42	0	32	0		0		0
105	0	1	1	0	0	34	0	33	0		0		0
106	0	6	1	0	0	39	0	36	0		0		0
107	0	14	1	0	0	37	0	39	0		0		0
108	0	14	1	0	0	73	0	32	0		0		0
109	0	15	1	0	0	1423	0	392	1	1583	0		1
110	0	15	1	0	0	36	0	34	0		0		0

111	0	13	1	0	0	33	0	2147	1	27	0		1
112	0	13	1	0	0	35	0	33	0		0		0
113	0	15	1	0	0	31	0	29	0		0		0
114	0	6	1	0	0	39	0	57	0		0		0
115	0	0	1	0	0	212	0	183	0		0		2
116	0	0	1	0	0	428	0	370	0		0		1
117	0	9	1	0	0	69	0	50	0		0		0
118	0	9	1	0	0	510	0	716	0		0		1
119	0	9	1	0	0	54	0	89	0		0		0
120	0	9	1	0	0	194	0	133	0		0		2
121	0	0	1	0	0	111	0	81	1	111	0		2
122	0	13	1	0	0	41	0	28	0		0		0
123	0	11	1	0	0	146	0	675	1	133	0		1
124	0	11	1	0	0	204	0	294	1	74	0		2
125	0	11	1	0	0	128	0	106	0		0		2
126	0	15	1	0	0	51	0	164	1	32	0		0
127	2	6	1	0	0	172	0	160	0		0		2
128	2	0	1	0	0	330	0	136	1	101	0		2
129	2	2	1	0	0	245	0	140	0		0		2
130	2	7	1	0	0	230	0	337	1	110	0		2
131	2	0	1	0	0	542	0	136	1	56	0		2
132	2	0	1	0	0	240	0	753	1	218	0		1
133	2	0	1	0	0	728	0	378	0		0		1
134	2	0	1	0	0	1300	0	500	0		0		1
135	2	6	1	0	0	339	0	287	0		0		1
136	2	1	1	0	0	201	0	309	1	86	0		2
137	2	1	1	0	0	385	0	356	0		0		1
138	2	2	1	0	0	444	0	495	0		0		1
139	2	2	1	0	0	259	0	270	0		0		1
140	2	2	1	0	0	145	0	263	1	99	0		2
141	2	2	1	0	0	118	0	301	1	60	0		2
142	2	2	1	0	0	716	0	317	0		0		1
143	2	1	1	0	0	382	0	263	0		0		1
144	2	1	1	0	0	221	0	254	1	100	0		2

145	2	6	1	0	0	1835	0	480	0		0		1
146	2	6	1	0	0	154	0	131	0		0		2
147	2	0	1	0	0	427	0	518	0		0		1
148	2	0	1	0	0	1485	0	1524	0		0		1
149	2	0	1	0	0	709	0	979	0		0		1
150	2	0	1	0	0	172	0	154	0		0		2
151	2	0	1	0	0	124	0	355	1	107	0		2
152	2	8	1	0	0	81	0	58	0		0		0
153	2	8	1	0	0	60	0	36	0		0		0
154	2	8	1	0	0	199	0	43	1	41	0		0
155	2	0	1	0	0	106	0	35	1	40	0		0
156	2	0	1	0	0	41	0	32	0		0		0
157	2	0	1	0	0	346	0	33	1	42	0		2
158	2	2	1	0	0	268	0	74	1	126	0		2
159	2	2	1	0	0	80	0	122	1	55	0		0
160	2	9	1	0	0	38	0	52	0		0		0
161	2	0	1	0	0	65	0	887	1	77	0		1
162	2	0	1	0	0	75	0	69	0		0		0
163	2	0	1	0	0	149	0	77	1	63	0		0
